# Supplementary material for: Highly stretchable carbon aerogels
Source: Nat Commun. 2018 Feb 28;9:881. doi: 10.1038/s41467-018-03268-y (PMC5830400; doi:10.1038/s41467-018-03268-y)
Supplement: Supplementary file 1 — Supplementary Information [file 41467_2018_3268_MOESM1_ESM.pdf]

Supplementary Information

Highly Stretchable Carbon Aerogels

Fan Guo[1]†, Yanqiu Jiang[1]†, Zhen Xu[1]*, Youhua Xiao[1], Bo Fang[1], Yingjun Liu[1], Weiwei Gao[1], Pei Zhao[2], Hongtao Wang[2], Chao Gao[1]*

[1]MOE Key Laboratory of Macromolecular Synthesis and Functionalization, Department of Polymer Science and Engineering, Zhejiang University, 38 Zheda Road, Hangzhou 310027, P. R. China

[2]Institute of Applied Mechanics and Key Laboratory of Soft Machines and Smart Devices of Zhejiang Province, Zhejiang University

*Corresponding author: zhenxu@zju.edu.cn (Z.X.); chaogao@zju.edu.cn (C.G.)

† These authors contributed equally to this work

Supplementary Table 1 | Comparison of materials, density, maximum tensile strain and maximum cycle numbers of ultralight stretchable aerogels in this work and previous reports. As a result of the pre-buckling hierarchy methodology and synergistic effect, the optimized carbon aerogels deliver high stretchability and good fatigue resistance at the same time compared to the state-of-the-art ductile aerogels with low density.

Ref.	Material	Density ($mg\ cm^{-3}$)	Maximum tensile strain	Maximum cycle numbers	Method
**Our work**	**bCAs**	**5.7**	**200%**	**1,000,000**	**hierarchical synergistic assembly**
**30**	Graphene/CNT/PDMS	-	85%	5000	Introduction of elastomeric materials as filling or substrate
**31**	CNT/ rubber fiber	-	1320%	200,000	
**32**	CNT/PDMS	-	100%	20	
**33**	CuNF/PDMS	10	60%	1000	
**34**	CNT	4	25%	1000	Percolation networks
**35**	Cellulose/carbon nanotubes	150–170	6%	none	
**36**	Nanofibre-assembled cellular	9.6	7%	none	
**37**	Cellulose nanofibril/SWNT	1500	8%	2900	
**38**	Ni	2	20%	none	Additive manufacturing
**39**	Alumina/polymer	200	4.5%	none	

Supplementary Movie 1. *In-situ* stretching-retraction video of a 30% MWNT@G bCA to ~100% strain. The bCA demonstrates nearly complete recovery after stretched.

Supplementary Movie 2. *In-situ* TEM observation of a binary laminate in a single stretching-retraction cycle for ~700 nm.

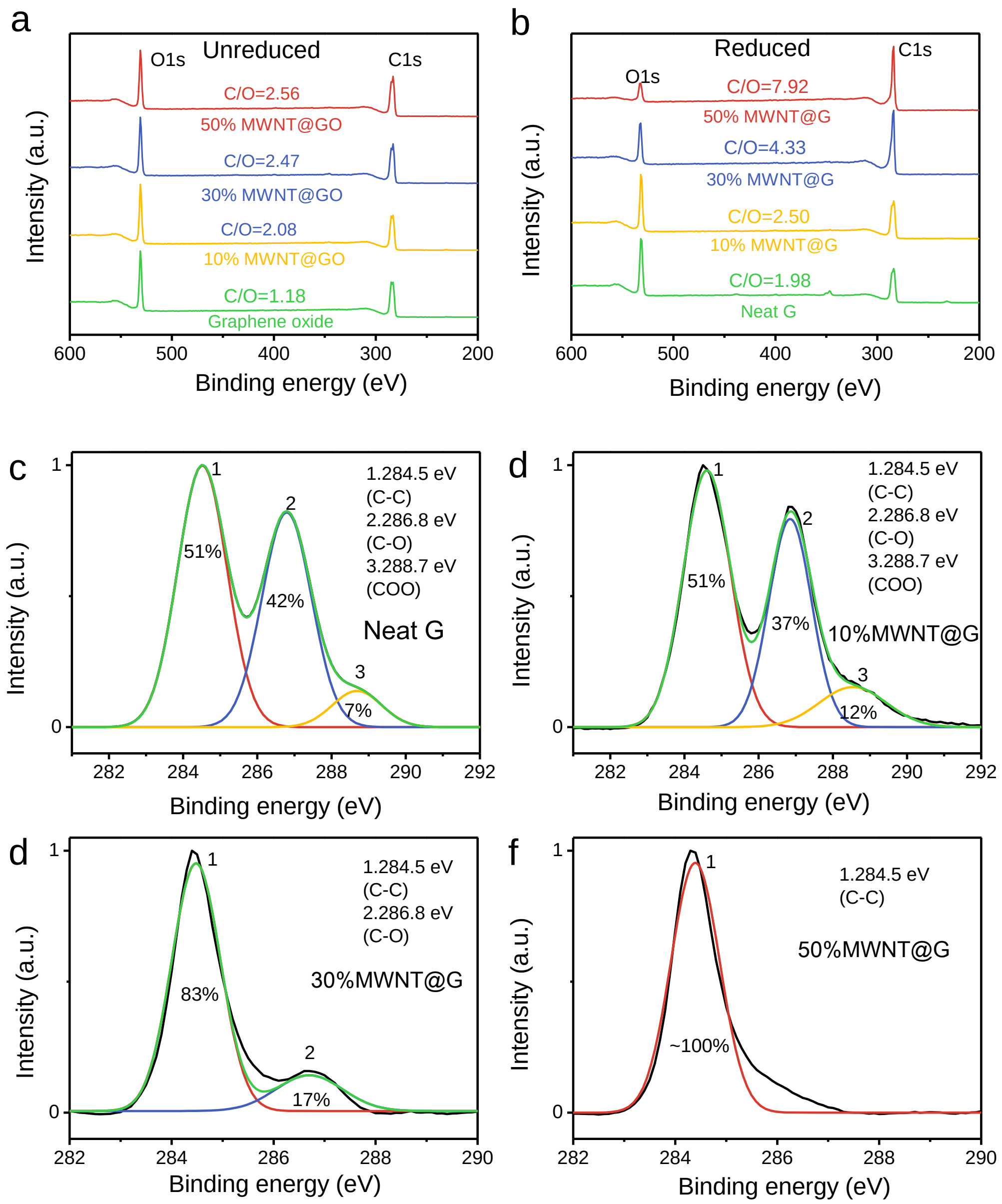

Supplementary Figure 1 | XPS spectra of bCAs. a, XPS spectrum of GO-MWNT aerogel with different MWNTs doses. **b**, XPS spectrum of rGO-MWNT aerogel with different MWNTs doses. **c-f**, High-resolution XPS spectra for C 1s of rGO-MWNT aerogel with different MWNTs from (**c**) 0% (**d**) 10% (**e**) 30% to (**f**) 50%.

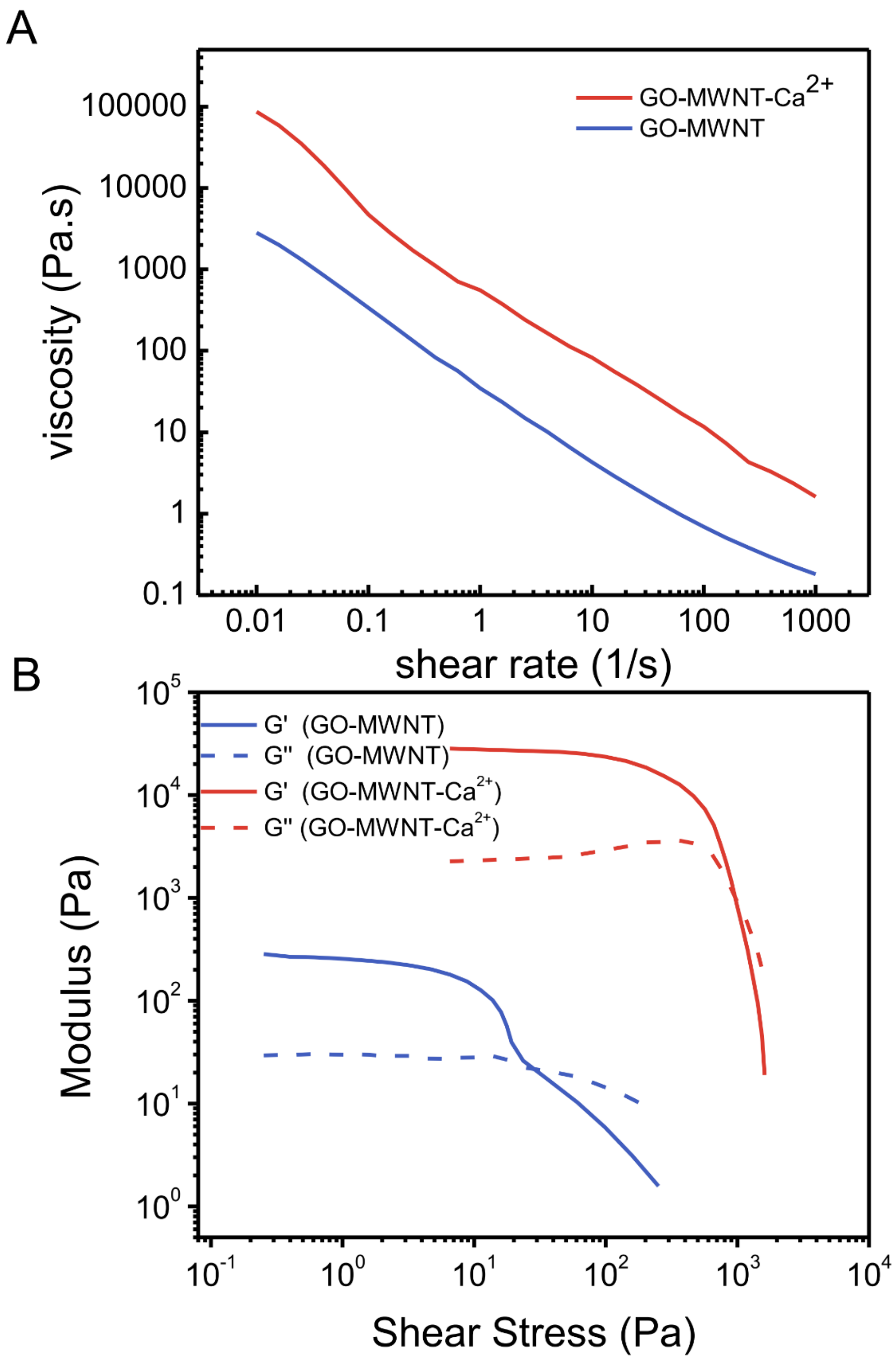

Supplementary Figure 2 | Rheological behavior of printable GO-MWNT inks. a, Viscosity as a function of shear rate. **b**, The storage and loss modulus as a function of shear stress.

The addition of Ca^{2+} favored the ink with a steep decreasing elastic modulus under high shear stress, which makes the ink fluxible in the nozzle, and a high static elastic modulus, which ensure the ink maintaining the original structure once extruded out of the nozzle.

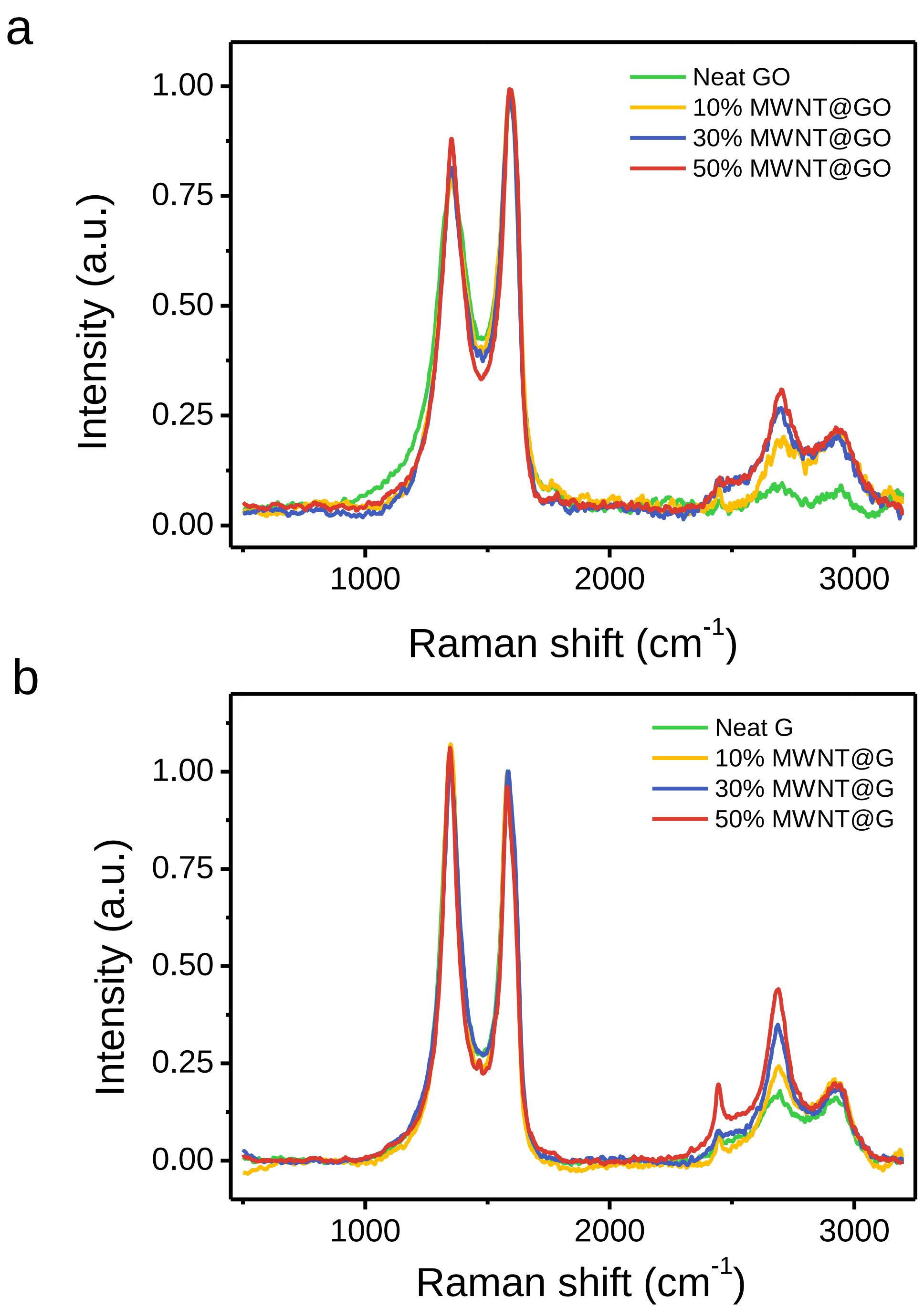

Supplementary Figure 3 | Raman spectra of bCAs. a, Raman spectra of GO-MWNT aerogel with different MWNTs doses. **b**, Raman spectra of rGO-MWNT aerogel with different MWNTs doses.

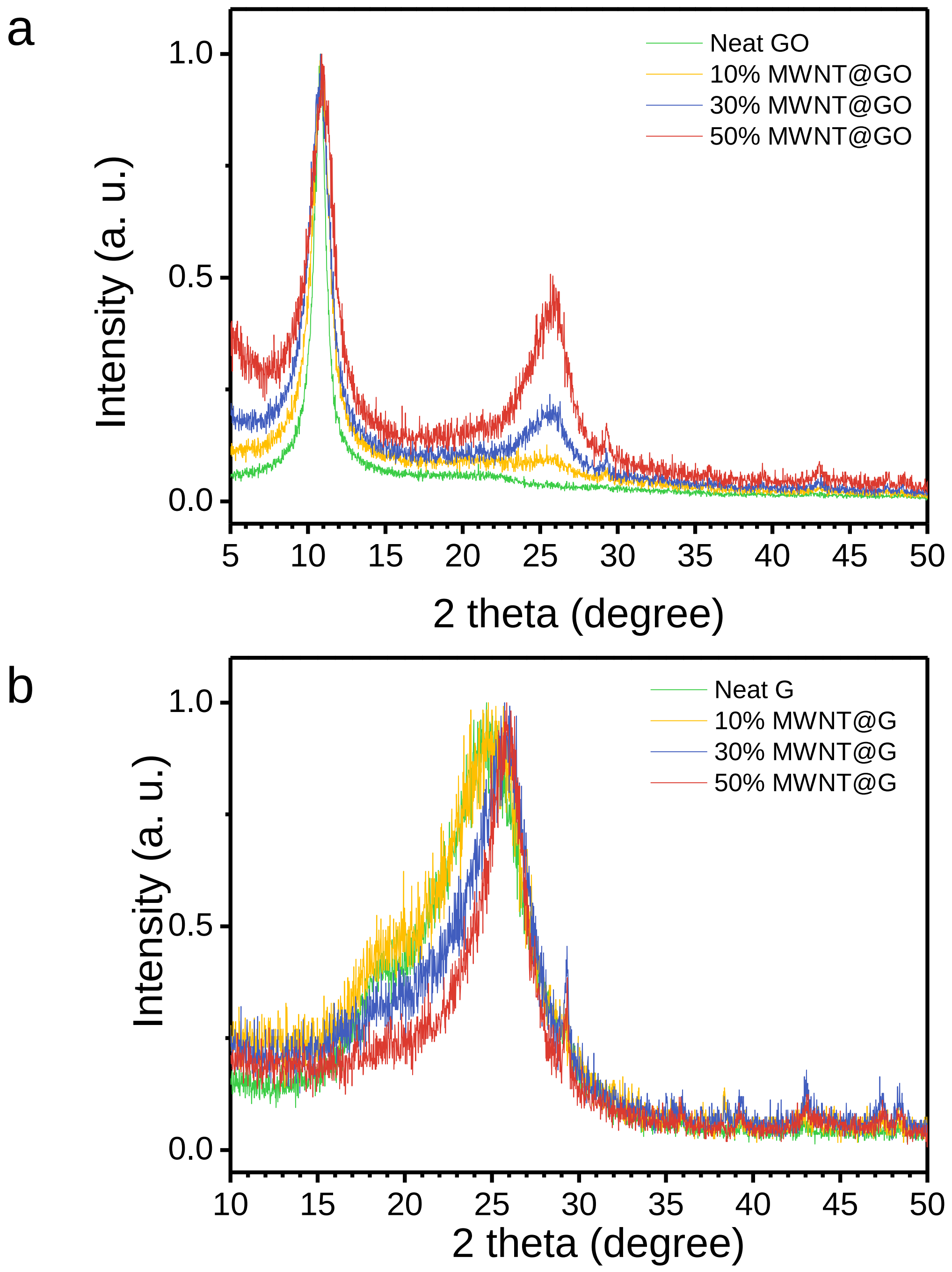

Supplementary Figure 4 | XRD patterns of bCAs. a, XRD patterns of GO-MWNT aerogel with different MWNTs doses. **b**, XRD patterns of rGO-MWNT aerogel with different MWNTs doses.

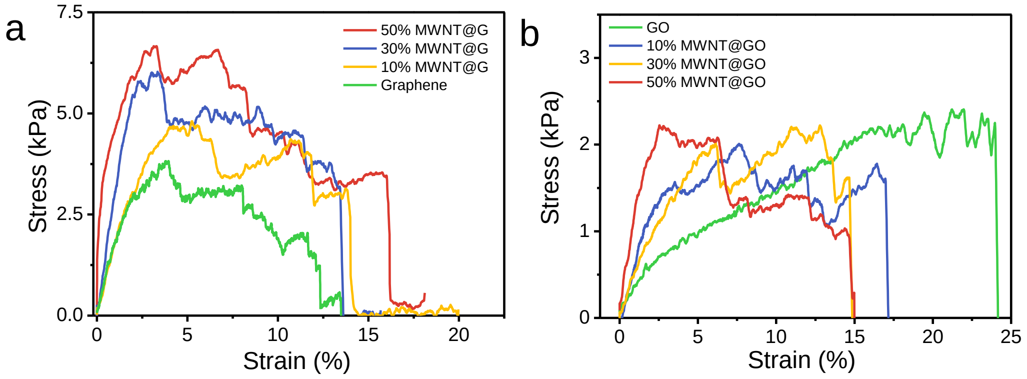

Supplementary Figure 5 | Stress-strain curves of reduced and unreduced single beams. a, Reduced binary carbon beams emerged higher breaking strength than pure graphene beams. **b**, Unreduced pure graphene beams showed highest (~24%) elongation..

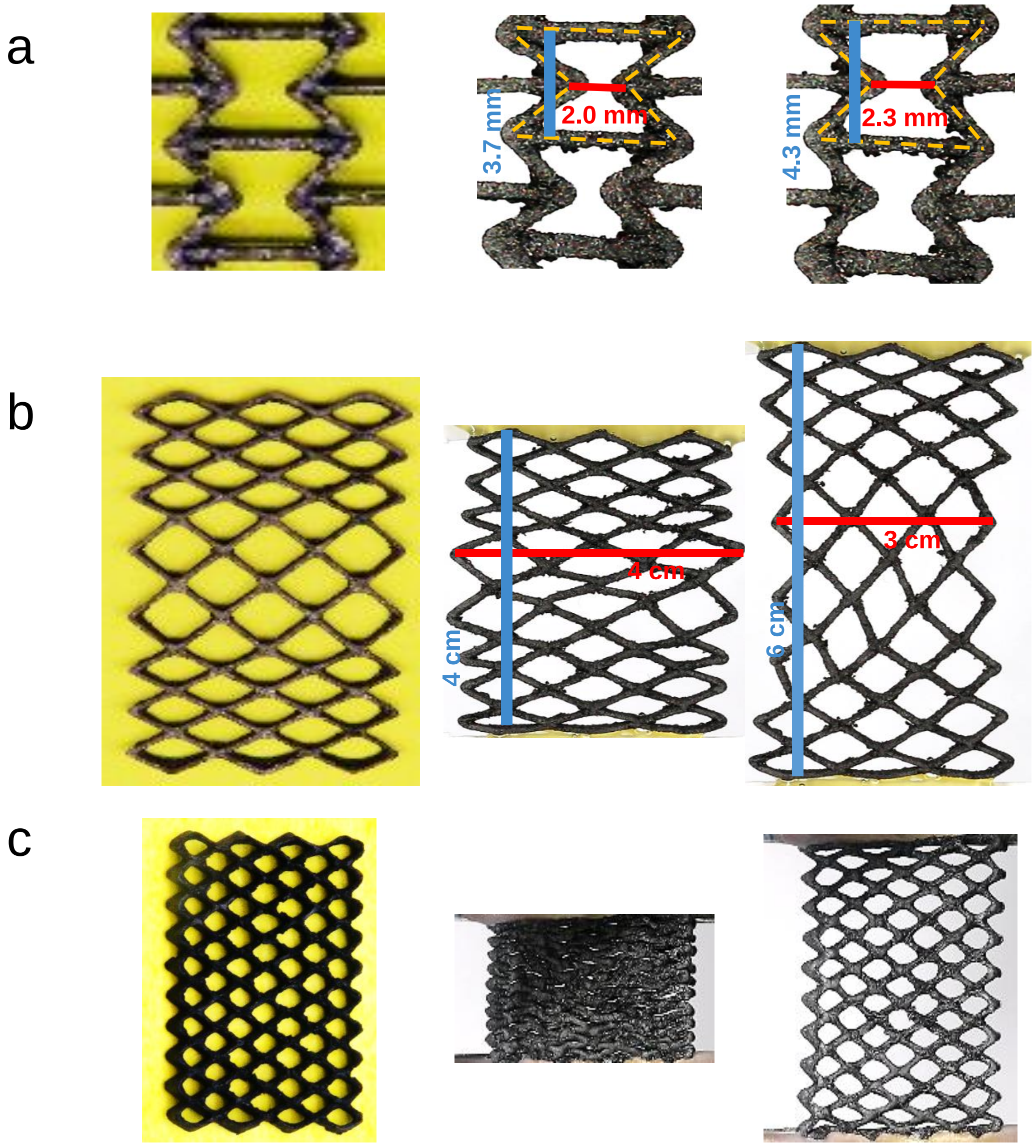

Supplementary Figure 6 | Various programmed lattices with different Poisson ratios. a, Negative Poisson ratio (re-entrant honeycomb). **b**, Positive Poisson ratio. **c**, Nearly zero Poisson ratio. The binary carbon aerogels based on graphene cell walls coated with MWNTs show a negative Poisson's ratio. By designing different base patterns, we achieved highly stretchable bCAs with different Poisson ratios. The re-entrant honeycomb is an auxetic geometry and the distance (red line in **a**) increased from 2.0 mm to 2.3 mm under 10% strain. The rhombus broadens under compression while the polygon cells (2nd order) show negative alert in volume. This positive alerting geometry together with negative alerting materials collectively composes bCA with near-zero Poisson ratio.

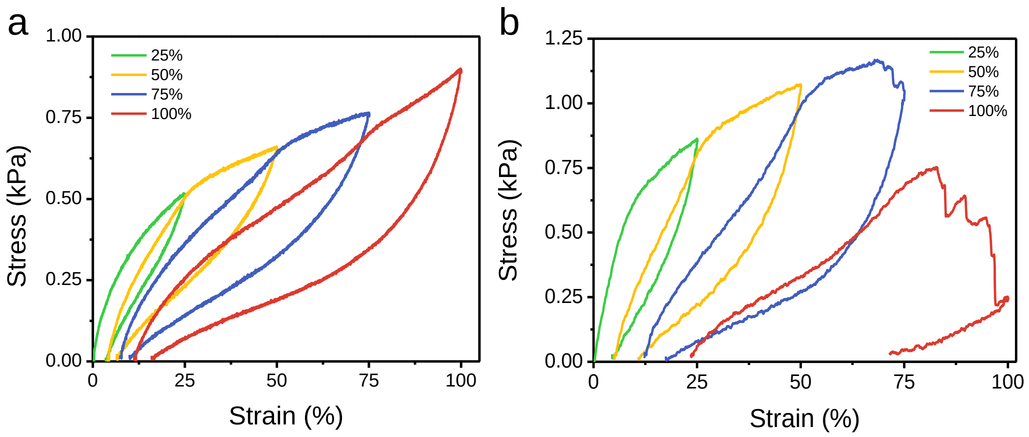

Supplementary Figure 7 | Typical stress-strain curves of unstable neat graphene aerogels. a, The best tensile-recovery performance of neat graphene aerogel. **b**, The worst tensile-recovery performance of neat graphene aerogel. Because the brittle graphene sheets catastrophically propagate cracks once initiate, the tensile curves of various samples are widely variant. We chose the best performance of neat GA as blank control samples as shown in Figure 2 in the main text.

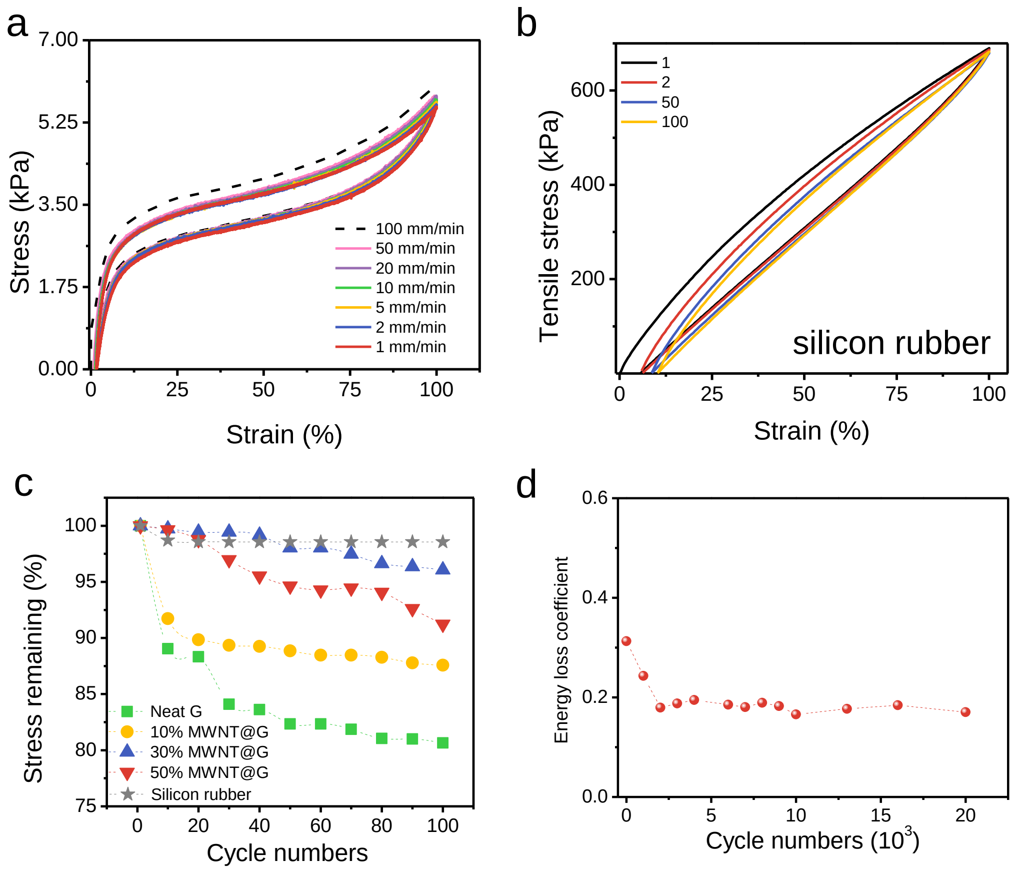

Supplementary Figure 8 | Mechanical properties of the optimized bCA with 30 wt. % MWNTs. a, The frequency independence of stress-strain curves of bCA by different test rates ranging from 1mm/min to 100 mm/min. **b**, Stress-strain curves of silicon rubbers. **c**, Stress remaining of bCAs with different doses during 100% cyclic tensile strain. **d**, Energy loss coefficient of bCA at 25% strain for 20,000 cycles.

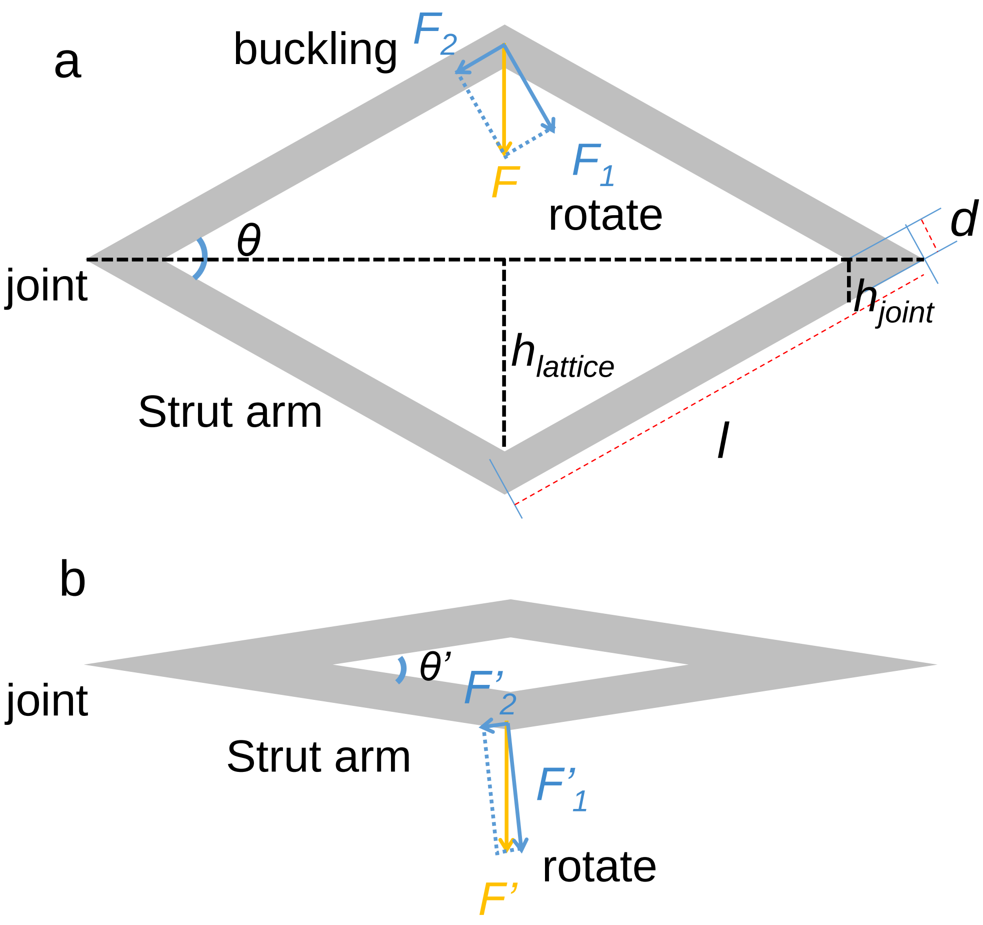

Supplementary Figure 9 | Geometrical and force analysis of rhombus millimeter lattices. a and **b**, Amplify ratio (σ) between the local deformation of joint and the rhombus millimeter lattice is given by:

$$\sigma = \frac{h_{lattice}}{h_{joint}} = \frac{l \cdot \sin\frac{\theta}{2}}{d/cos\frac{\theta}{2}} = \frac{l}{2d} \cdot \sin\theta \quad (1)$$

And β (stretchable ratio) is given by $\beta = f(\sigma) + f(d)$, When the angel (θ) is determined, β is proportional to *l/d*. The component force F_1 is perpendicular to the strut arm and therefore causes the arms rotate about the joint. Another component force F_2 is parallel to the arm and causes the buckling of truss arms.

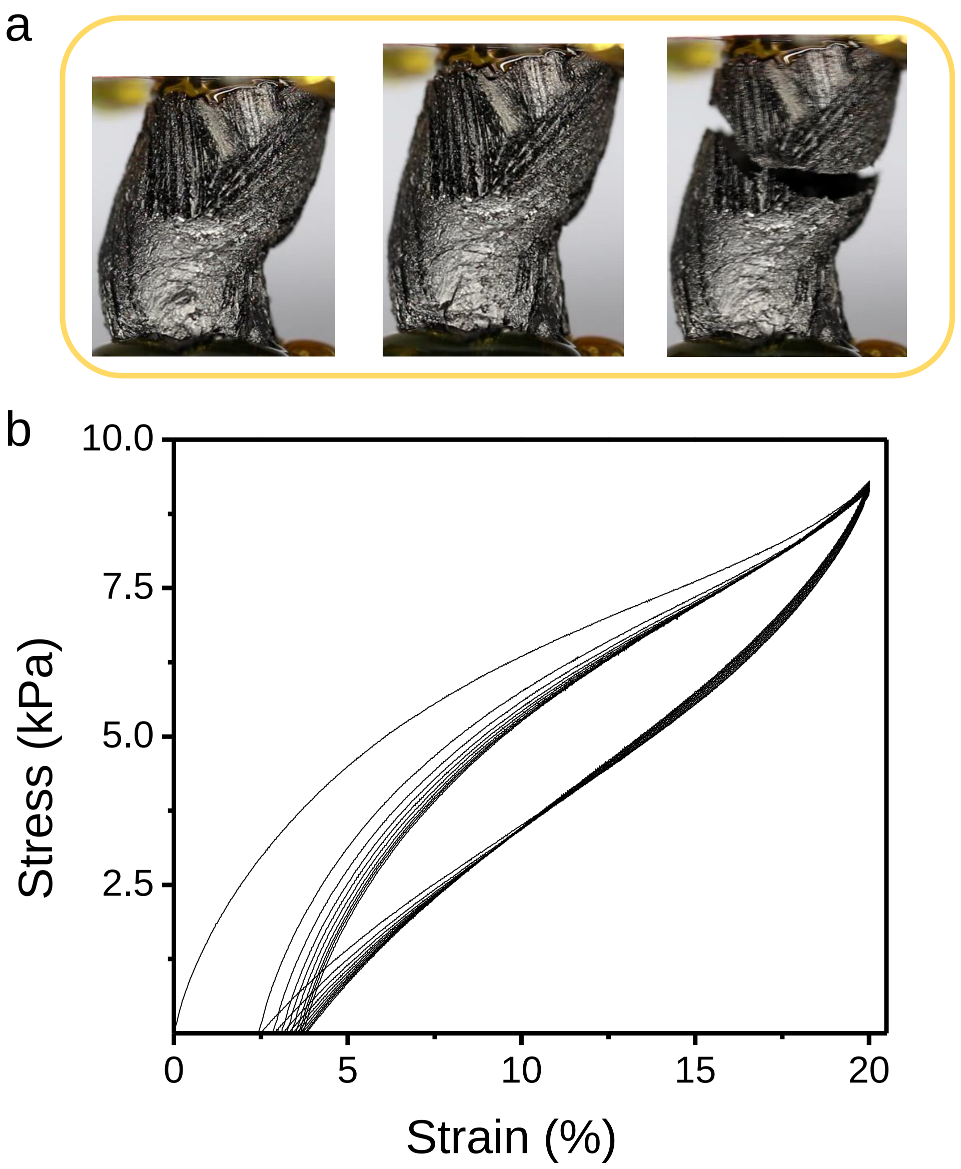

Supplementary Figure 10 | Mechanical test of bulk CAs. a, Images of bulk aerogel (height: 10mm, diameter: 6.5mm) under tensile stress. **b**, Relative stress-strain curves. Bulk CAs demonstrated stretching strain ~20% but no border platform in the curve. It is the interact beams and nodes in truss structure that impede abrupt structure destabilization and allow gradually transformation to new equilibrium state, resulting in the border plateau in the stress-strain curve and large elongation.

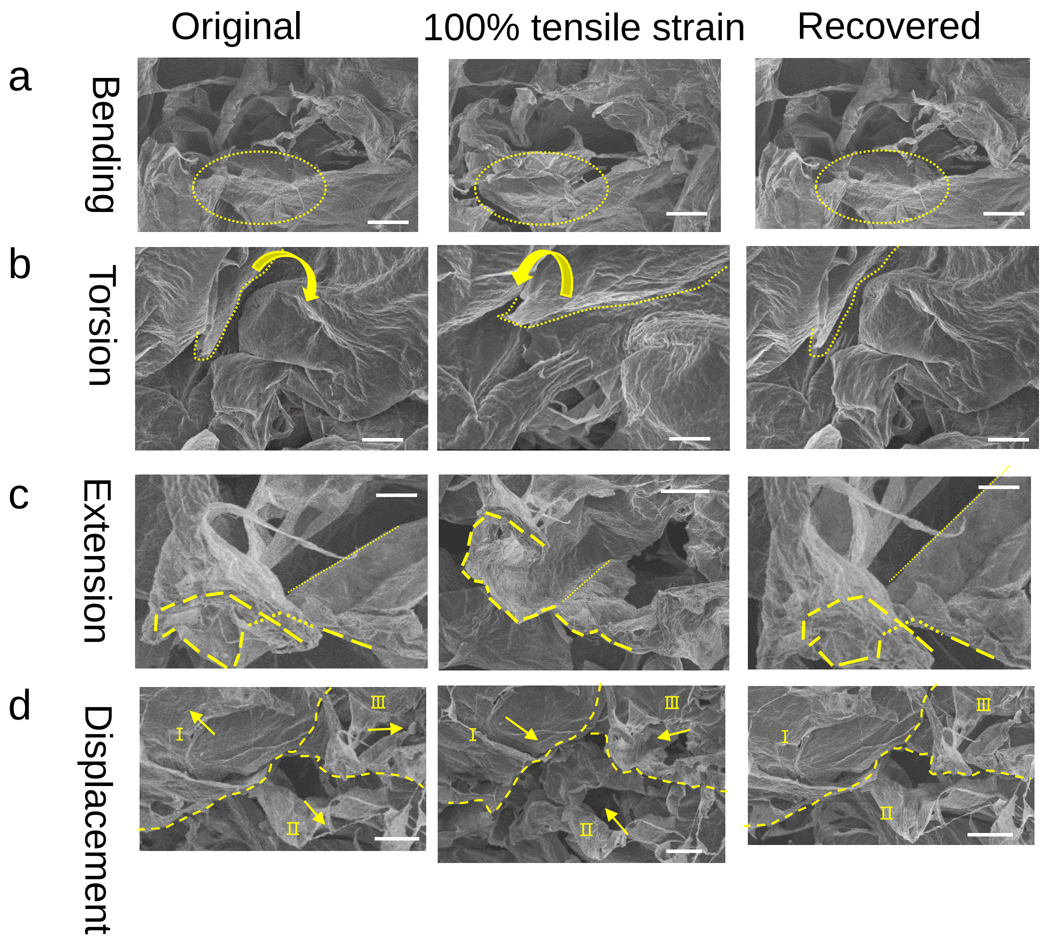

Supplementary Figure 11 | Deformation modes of cell walls under tensile load. **a-d**, Bending, torsion, extension and displacement in a cycle. Scale bars: 15 μm, 3 μm, 10 μm and 20 μm from **a** to **d**.

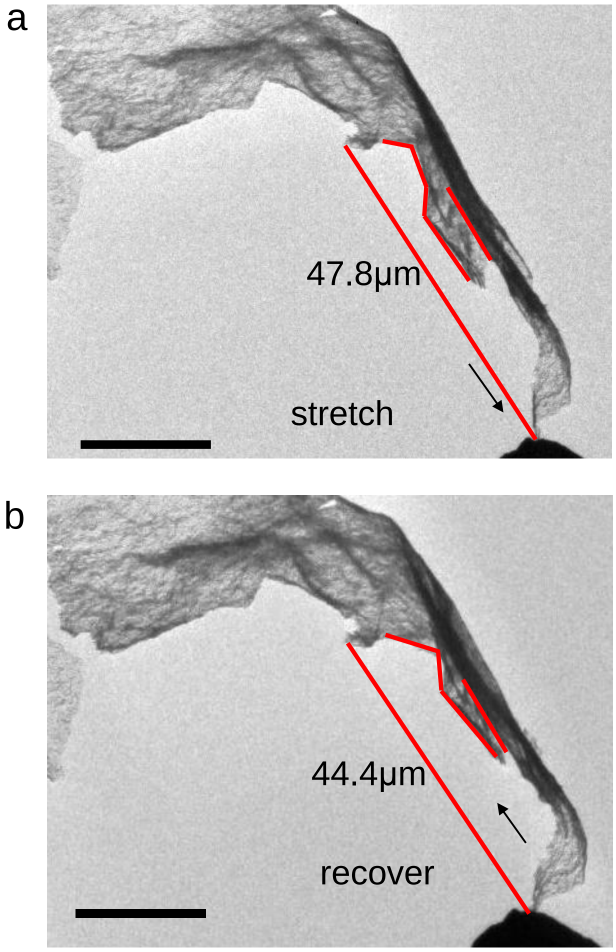

Supplementary Figure 12 | *In-situ* TEM images of a single synergistic flake. a, stretched. **b**, recovered. Transmission electron microscopy (TEM) revealed that stretching and retraction of single synergistic flake to verify the results from AFM. It is hard to guarantee the force across the barycenter of flake, as a result, torque generated torsion together with extension of flake. This torsion is completely reversible, for the shape of synergistic flake is exactly the same as original shape after stretching and compressing. The complete deformation process can be seen in supplementary movie 2. Scale bars, 10μm (**a** and **b**)

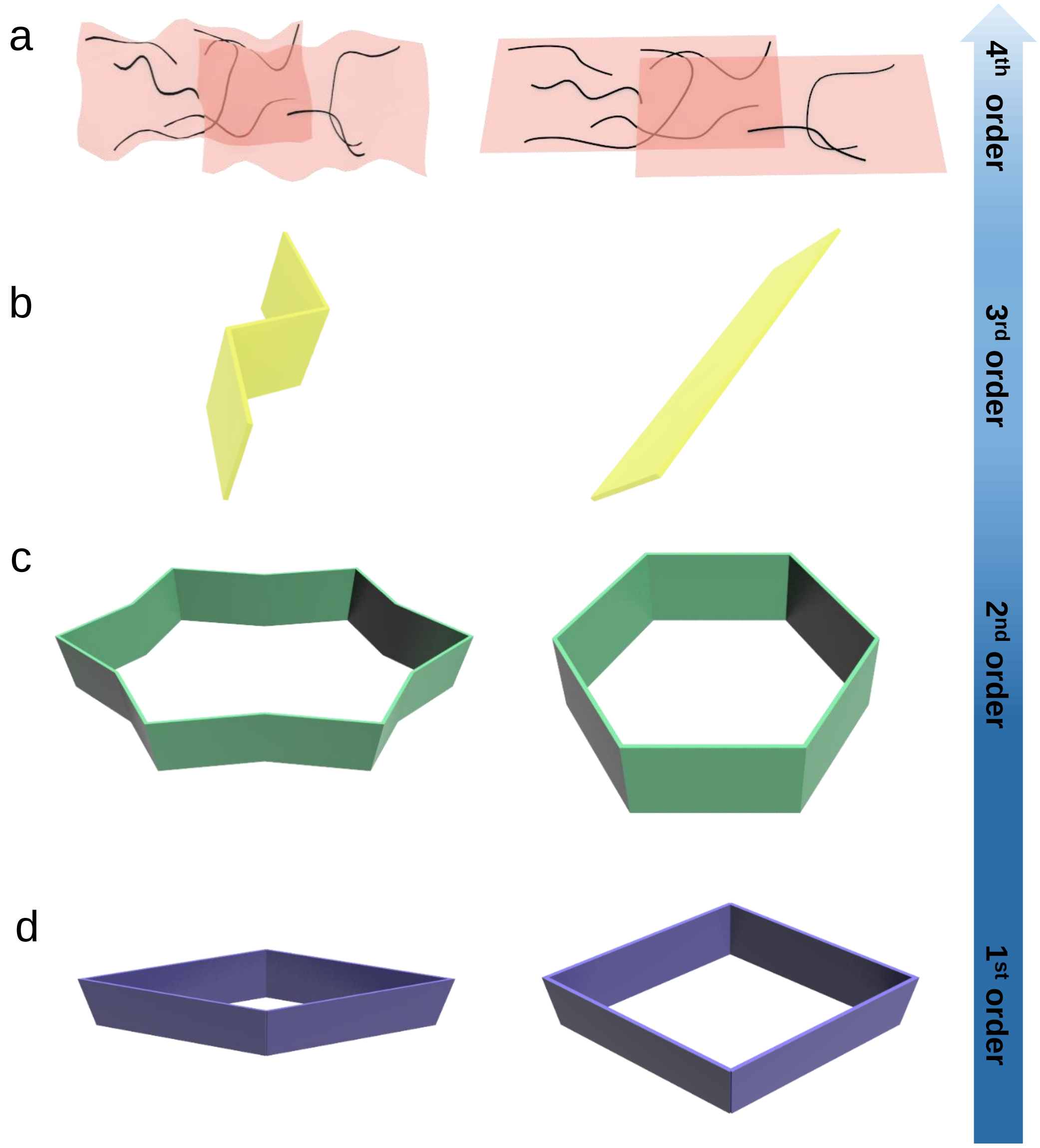

Supplementary Figure 13 | Schematic illustration of the multi-order deformation process.

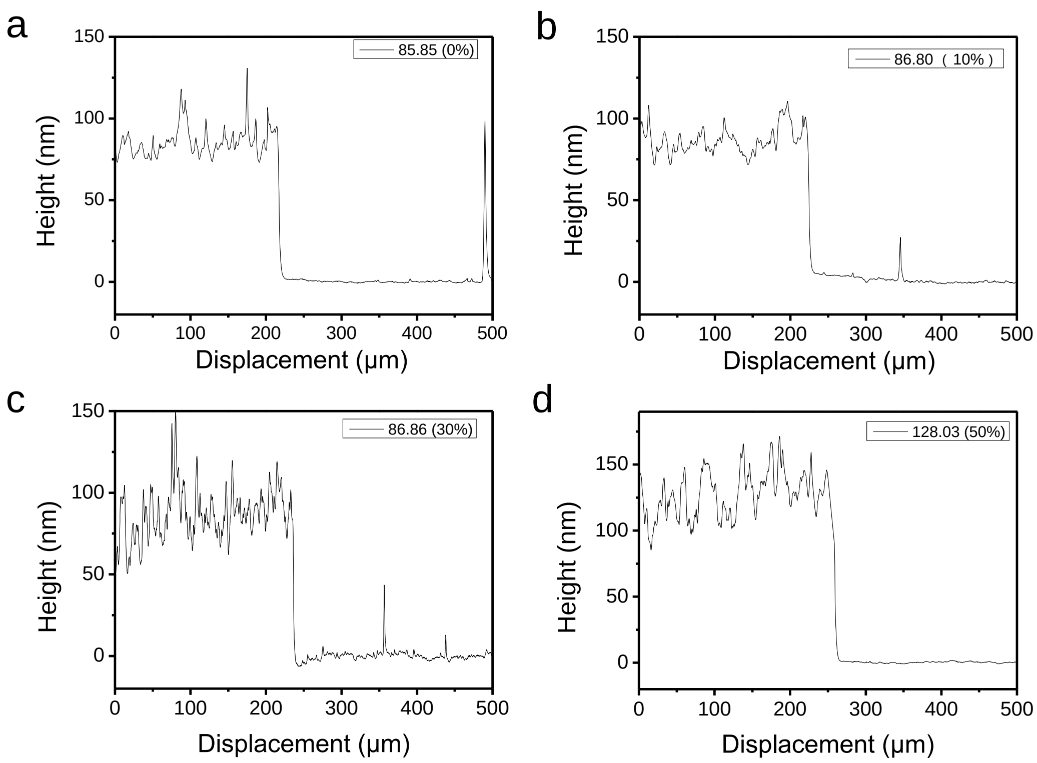

Supplementary Figure 14 | The thickness of thin binary carbon composite films measured by step profiler. a-d, Thickness of 0%, 10%, 30%, 50% rGO-MWNT films. The fluctuations stem from the wrinkles and surface roughness.

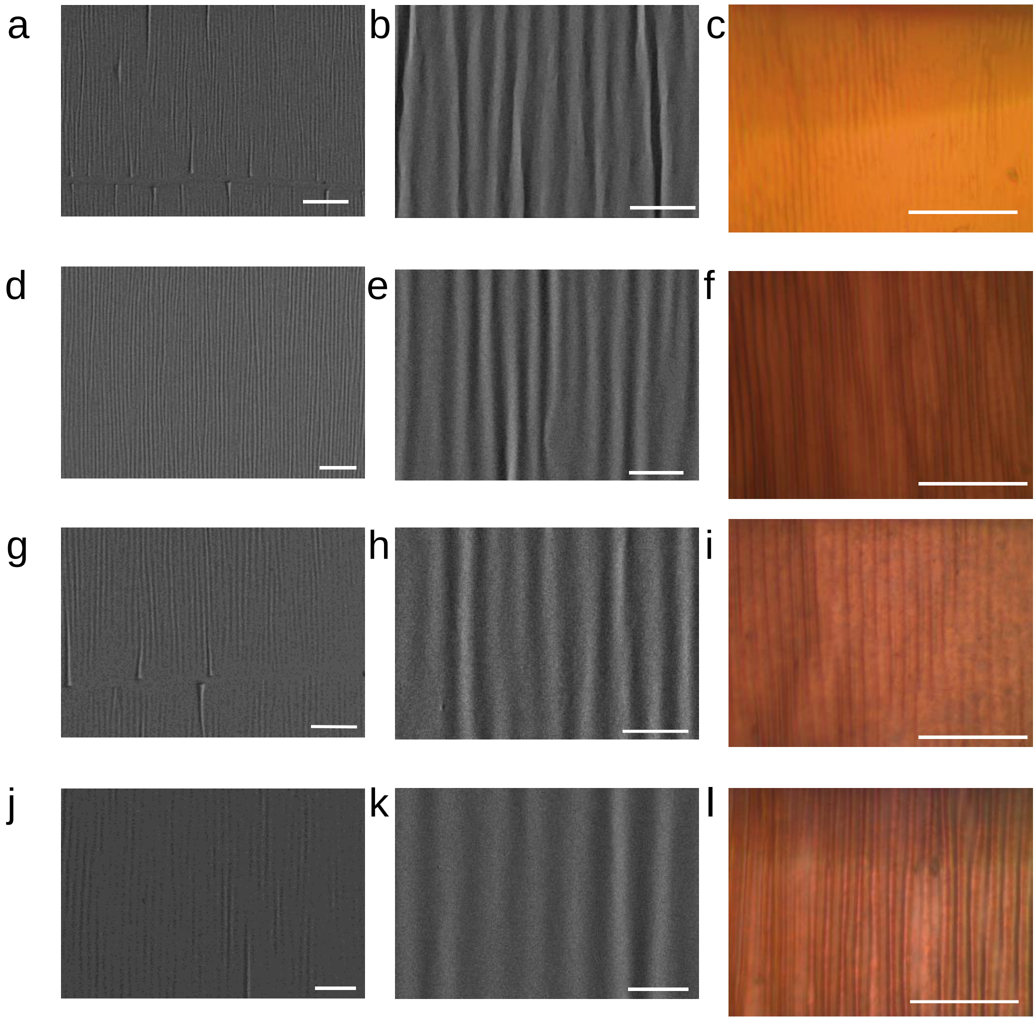

Supplementary Figure 15 | SEM and optical images of wrinkles. a-l, Wavelength of 0 wt.%, 10 wt.%, 30 wt.%, 50 wt.% rGO-MWNT film. Scale bars, 100µm (**a, d, g, j**), 20µm (**b**, **e**, **h**, **k**), 200µm (**c**, **f**, **i**, **l**)

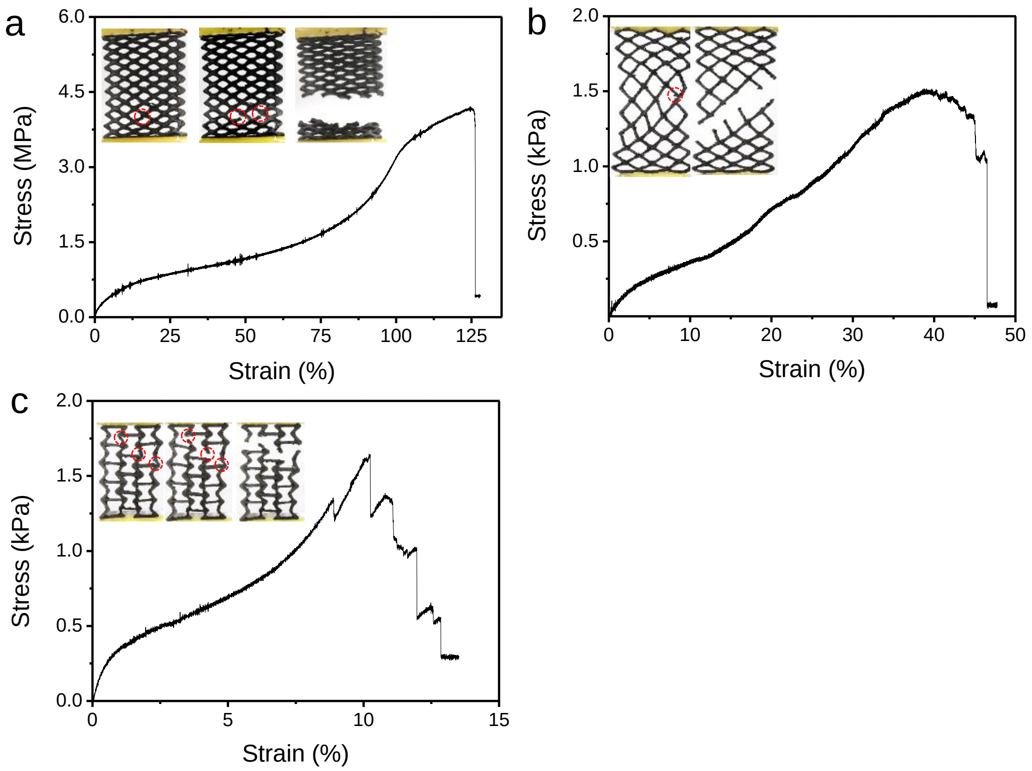

Supplementary Figure 16 | The critical fracture curves of bCAs. a-c, Beams composing the truss lattice ruptured one-by-one under high tensile stress and ultimately lead to the totally breakage of monolithic bCA. The stress gradually stepped down until bCA was completely torn instead of abruptly linear descent.

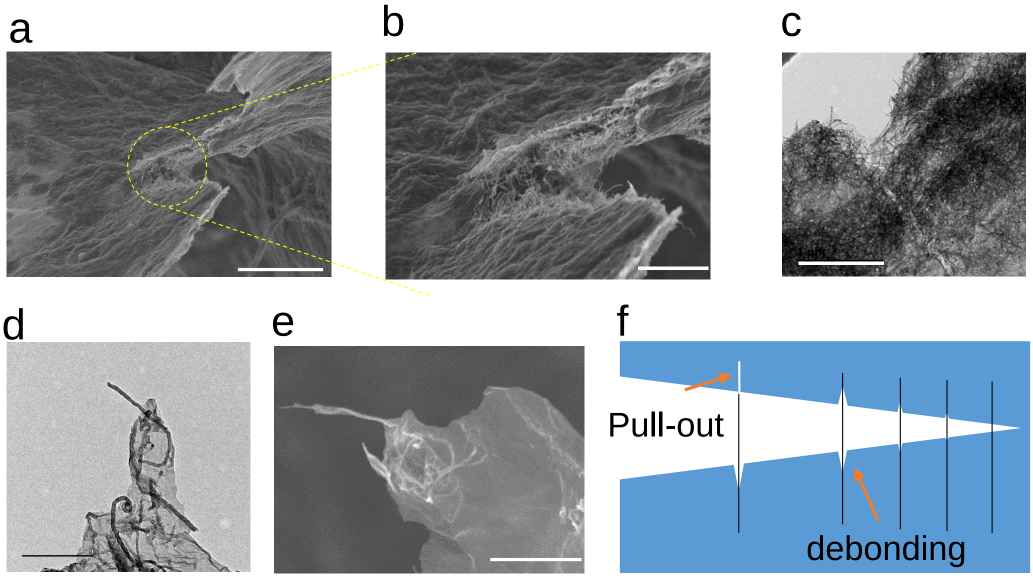

Supplementary Figure 17 | a-c, Images of crack at graphene layers sandwiched by intertwined MWNTs. **d-f**, The synergistic effect was undermined to some degree by means of debonding and pull-out of MWNTs from graphene sheets under cyclic stress. The destroyed synergistic effect can no longer suppress the cracks propagation and resulting the structural failure. Scale bars, 5 μm (**a**), 2 μm (**b**), 1μm (**c**), 500 nm (**d**) and 1μm (**e**).

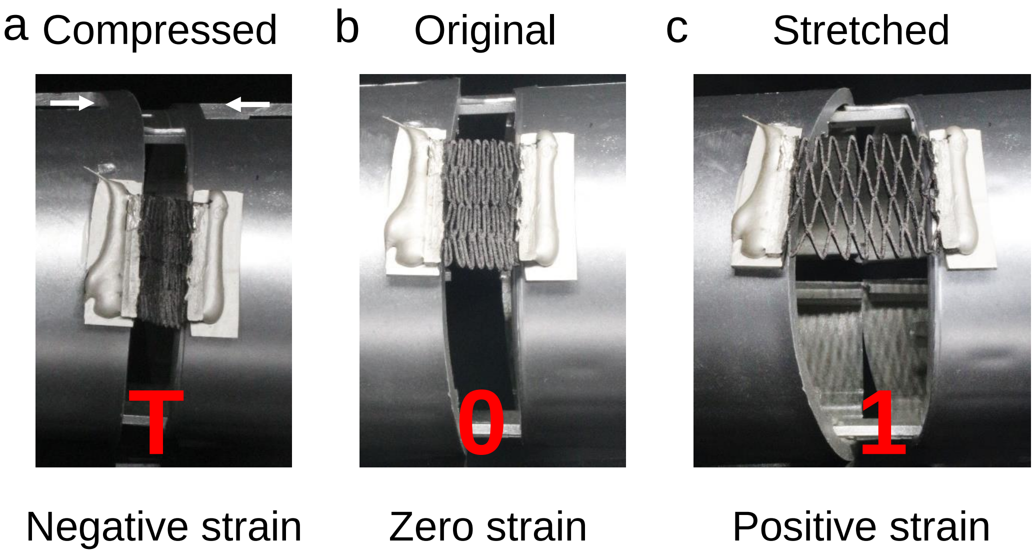

Supplementary Figure 18 | Images of bCAs as logical sensors at joints.
