# Supplementary material for: Highly stretchable carbon aerogels
Source: Nat Commun. 2018 Feb 28;9:881. doi: 10.1038/s41467-018-03268-y (PMC5830400; doi:10.1038/s41467-018-03268-y)
Supplement: Supplementary file 3 — Description of Additional Supplementary Files [file 41467_2018_3268_MOESM3_ESM.pdf]

Description of Additional Supplementary Files

File Name: Supplementary Movie 1
Description: *In-situ* stretching-retraction video of a 30% MWNT@G bCA to ~100% strain.

File Name: Supplementary Movie 2
Description: *In-situ* TEM observation of a binary laminate in a single stretching-retraction cycle for ~700 nm.
